# Nematocidal Activity of Ethanol and Aqueous Extracts of *Persea americana* Seeds against *Heligmosomoides polygyrus* using the Worm Microtracker Method

**DOI:** 10.1155/2023/9545565

**Published:** 2023-09-14

**Authors:** Yamssi Cédric, Noumedem Anangmo Christelle Nadia, Sandra Nfufu, Mounvera Abdel Azizi, Tientcheu Noutong Jemimah Sandra, Vincent Khan Payne

**Affiliations:** ^1^Department of Biomedical Sciences, Faculty of Health Sciences, University of Bamenda, P.O. Box 39 Bambili, Cameroon; ^2^Department of Microbiology, Haematology and Immunology, Faculty of Medicine and Pharmaceutical Sciences, University of Dschang, P.O. Box 96 Dschang, Cameroon; ^3^Department of Medical Laboratory Sciences, Faculty of Health Sciences, University of Bamenda, P.O. Box 39 Bambili, Cameroon; ^4^Department of Animal Biology, Faculty of Science, University of Dschang, P.O. Box 067 Dschang, Cameroon

## Abstract

**Background:**

Infections with gastrointestinal helminths constitute a serious obstacle to the good health of the local population in most African Countries. The aim of this study was to evaluate the anthelminthic activity of *Persea americana* ethanol and aqueous extracts against *Heligmosomoides polygyrus* using the worm microtracker.

**Method:**

Aqueous and ethanolic extracts of *P. americana* were prepared. Different concentrations of the extracts were tested against the egg and larvae stages of *H. polygyrus* using an automated high-throughput method. Briefly, embryonated eggs and larvae of this parasite were obtained after the incubation of fresh eggs at 25°C for 24, 48, and 96 hours for embryonated eggs, L_1_ and L_2_ larvae, respectively. One hundred microliters of the plant extracts at various concentrations were put in contact in a 96-well microplate with a suspension of 100 embryonated eggs in a total volume of 200 *μ*L and incubated in a worm microtracker where the motility of the worms was recorded every 30 minutes for the ovicidal activity. The final tested extract concentration was 5, 2.5, 1.25, 0.625, and 0.3125 mg/mL, whereas ringer solution (0.95%) and 1.5% Dimethyl sulfoxide (DMSO) were used as negative controls and levamisole as positive control. The same method was used for larvicidal activities. The anthelmintic activity was determined using the average movement of the worms in the tested product compared with the negative control (1.5% DMSO and ringer solution).

**Results:**

The egg hatching rates of *H. polygyrus* had IC_50_ of 0.49 mg/mL (95% confidence interval: 71.70–92.03) and 0.22 mg/mL (95% confidence interval: 74.28–86.18) for the ethanol and aqueous extract, respectively. These IC_50_ indicate that the aqueous extract is more active for the inhibition of hatching at a 95% confidence interval. The aqueous and ethanol extracts presented mean inhibitory hatching rates of 78.33 ± 1.67% and 75.67 ± 1.15% at 5 mg/mL, respectively, with no significant differences. The highest percentage of inhibition of L_1_ larva was observed at 5 mg/mL with 89 ± 2.3%and 85 ± 2.7% for the ethanol and aqueous extracts, respectively. The lowest percentage of inhibition was observed at 0.3125 mg/mL, with 54.67 ± 3.38% and 49 ± 2.64% for the ethanol and aqueous extract, respectively. No significant differences were observed between the two extracts at 5 mg/mL with an inhibitory percentage of 90.67 ± 3.05% (ethanol) and 89.33 ± 2.08% (aqueous).

**Conclusion:**

Extracts of *P. americana* seeds possess nematocidal activity, however, further *in silico* and *in vivo* investigations are necessary to confirm their anthelminthic activity.

## 1. Introduction

Helminthiases constitute a serious obstacle to the good health of the local population in most African Countries [1]. Furthermore, they are considered neglected tropical diseases partly because little attention is directed toward them, whereas more attention is directed toward other diseases, such as COVID-19, malaria, turberculosis, and Human immunodeficiency virus (HIV) [2]. These infections are responsible for great mortality and morbidity in humans and affect mostly school-age children by inhibiting their intellectual and growth rate rendering them vulnerable to other infections [3]. In African countries, helminthiasis is one of the neglected tropical diseases that need to be eradicated [4]. The study of Adoubryn et al. [5] presented a very high prevalence of parasite infection of 55.2% in Biankouma, Ivory Coast. In Cameroon, according to the National Program of Schistosomiasis and Gastrointestinal Helminth Control reported in 2006 that more than 10 million people were infected with various parasitic worms [6]. Cedric et al. [6, 7] demonstrated in the North West region of Cameroon a high prevalence of 23.5% among children in Bambili. The same observations were recorded by Igore et al. [8] in the Tonga Sub division, West Region of Cameroon. All this high prevalence indicates that Cameroon has a serious problem with helminthiases and is neglected in Cameroon despite all efforts being put in place by the government to eradicate this disease.

To control these parasitic infections, patients used synthetic anthelmintic drugs. However, unfortunately, the parasite has developed resistance to the available synthetic drugs [9]. These substances are also less available and sometimes have side effects. Some side effects have been reported and are equally toxic to the natural environment [10]. The search for newer sources of drugs to overcome this issue of resistance is now a global challenge preoccupying pharmaceutical companies and other research institutions [11]. This issue of resistance, poverty, low efficacy, and scarcity of synthetic drugs has drawn back the population, especially those living in tropical countries due to their dependence on natural plants to overcome helminthiasis [12].

Medicinal plants are often used by traditional healers in the western region of Cameroon to overcome this problem of resistance, high cost, and availability [13]. The misuse of synthetic drugs is now a serious challenge in the domain of drug discovery. It becomes imperative to screen local medicinal plant beliefs to possess anthelminthic activities by the local population to deeply study them and scientifically validate their usage in traditional medicine.

Avocado (*Persea americana*) peels, seeds, fruits, and leaves are commonly used in America and Africa for the treatment of hypertension, stomach pain, bronchitis, diarrhea, and diabetes [14]. Avocado seeds equally possess insecticidal, fungicidal, and antimicrobial activities [15]. Study shows that *P. americana* contains carbohydrates, proteins, vitamins, and phytochemicals like saponins, phenolics, alkaloids, tannins, and flavonoids, which contributes to its high nutritional and pharmaceutical habits [16]. Helminthiasis is treated in the western region of Cameroon with the help of *P. americana.*

Most anthelminthic *in vitro* tests are done on Petri dishes, lack repeatability, and are time-consuming [17].

The WMicroTracker (Phylumtech, Argentina), is an apparatus that measures the average motility of worms when in contact with a test product [18]. *Heligmosomoides polygyrus* mimics the infection of human parasites and is used as a model in anthelminthic drug discovery. Ngouateu et al. [19], Wabo et al. [10], and Komtangi et al. [20] used *Heligmosomoides bakeri* when evaluating the anthelminthic activity of some medicinal plants used in Cameroon. This study aimed to evaluate the anthelminthic activity of *P. americana* ethanol and aqueous extracts using the worm microtracker against *H. polygyrus* to deeply study them and scientifically validate their usage in traditional medicine.

## 2. Materials and Methods

### 2.1. Parasite

*Swiss albinos* mice were infected with L3 larva. After a prepatent period of 10 days, the infected mice started producing fresh eggs. The different stages of development of *H. polygyrus* were cultured on Petri dishes under ideal conditions, such as wet filter paper, following the method described by White [21]. *H. polygyrus* is a nematode used by pharmaceutical industries for the evaluation of anthelminthic compounds [22].

### 2.2. Collection and Identification of Plant Species

*P. americana* seeds, commonly known as avocado seeds, were bought from avocado sellers in Bamenda, and identified at the National Herbarium with the identification number 18604/sfr/Cam.

### 2.3. Preparation of Extracts

The method described by Wabo et al. [23] was used for the preparation of different extracts. Briefly, the seeds were grated using a kitchen grater and dry to obtain a powder. The plant powder (100 g) was introduced into 1 L of ethanol at 95% for three days and 1 L of boiled water (100°C) for 3 hours until the obtention of the filtrate. The filtrate was then dried at 50°C (drying at more than 50°C will denature the active principles in the extracts) to obtain the extract.

### 2.4. Production of Embryonated Eggs

The collection of eggs was done using the flotation technique described by Cédric et al. [13]. After the collection of fresh eggs, it was incubated at 25°C for 24 hours for the obtention of embryonated eggs and used for the evaluation of hatching.

### 2.5. Collection of *H. Polygyrus* L_1_ and L_2_ Larvae

Embryonated eggs were incubated 25°C in ringer solution for 24 and 72 hours for the obtention of L_1_ and L_2_ larvae, respectively.

### 2.6. Egg Hatching Test

The egg hatching test was evaluated using the worm microtracker method [13]. The final tested extract concentration ranged from 5 to 0.3125 mg/mL. Levamisole (5 *μ*g/mL) and 0.5% Dimethyl sulfoxide (DMSO) were used, respectively, as positive and negative controls in a 98-well microplate titer. The plates were incubated at 25°C for 24 hours in a worm microtracker where the movement of the new larvae were recorded every 30 minutes. The inhibition percentage was calculated as follows:

(1) $$\begin{array}{c} \%\text{Inhibition}=\frac{\text{Mobility activity of Control}-\text{msobility activity of the test sample}}{\text{Mobility activity of Control}}\times 100. \end{array}$$

### 2.7. Larvicidal Activity

The larvicidal activity was determined using the worm microtracker method described by Cédric et al. [13]. The L1 and L2 larvae were put in contact with extracts with the same concentrations as the ovicidal activity (5–0.3125 mg/mL) and incubated for 24 hours in a final volume of 200 *μ*L. The percentage of inhibition of larvae motility was determined [24].

### 2.8. Qualitative Phytochemical Screening

The method described by Harbone [25] was used to test for the presence of anthraquinones, anthocyanins, saponins, triterpenoids, sterols, and alkaloids.

### 2.9. Total Phenolic and Flavonoid Contents

The total phenolic and flavonoid contents were determined according to the method described by Sidiki et al. [26].

### 2.10. Ethical Considerations

The animals were well treated according to the guideline EEC Directive 86/609/EEC of the 24th November, 1986.

### 2.11. Statistical Analysis

The data was analyzed using the GraphPad Prism software version 8.0. The 50% inhibitory concentrations (IC 50) at a 95% confidence interval were determined using a standard curve obtained from the GraphPad.

## 3. Results

Table 1 shows the effect of *P. americana* extracts on the mean inhibitory hatching percentage and larvae motility. From the analysis of Table 1, the mean inhibitory hatching percentage of ringer solution and 1.5% DMSO were 0.00% (negative control) and 100% for levamisole (positive control). No significant difference was observed for the mean inhibitory hatching rate of the aqueous extract (78.33 ± 1.67%) and the ethanol extract (75.67 ± 1.15%) at 5 mg/mL.

The concentration of 5 mg/mL presented the highest percentage of inhibition for the L1 larva with 89 ± 2.3% (ethanol) and 85 ± 2.88% (aqueous). The inhibition was concentration-dependent. There was no significant difference in terms of their inhibition at the highest level of inhibition. Levamisole, which was the drug of choice (positive control), had the highest percentage (100.0%) of L_1_ larva. This value indicated that levamisole has an inhibitory effect on the L_1_ larva of *H. polygyrus*. For the L2 larva, the highest percentage of inhibition was observed at 5 mg/mL with 89.33 ± 1.2% (aqueous) and 90.67 ± 1.7% (ethanol). The lowest inhibition was observed at 0.3125 mg/mL, with 54.67 ± 5.8% and 49 ± 4.5% for the ethanol and aqueous extracts, respectively. From the different IC_50_ 0.49 mg/mL for the ethanol extract at 95% confidence interval (71.70–92.03) and 0.22 mg/mL for the aqueous extract at 95% confidence interval (74.28–86.18) it indicates that the aqueous extract is more active on egg hatching inhibition. A similar observation is noticed in the L1 and L2 larvae where the aqueous extract is more active than the ethanol extract.  Table 2 shows the phytochemical screening of the aqueous and ethanol extracts of Persea americana. It follows from the analysis of this table that the aqueous extract contains all the screen phytochemical constituent except sterols. Similarly, the ethanolic extract contains the same compounds except saponins and sterols.

### 3.1. Total Phenolic and Flavonoid Contents

More flavonoid content extract (453.9 ± 43.46 mg/g) was observed in the ethanol extract compared with the aqueous extract (174.54 ± 38.10 mg/g). The ethanol extract equally had more phenolic content (381.10 ± 9.23 mg/g) with respect to the aqueous extract (321.9 ± 11.14 mg/g).

## 4. Discussion

No significant difference was observed for the mean inhibitory hatching rate of the aqueous extract (78.33 ± 1.67%) and the ethanol extract (75.67 ± 1.15%) at 5 mg/mL. This mean hatching inhibition rate seen in the wells containing the extracts confirms the fact that *P. americana* seed extracts possess egg hatching inhibitory power. Wabo et al. [27] obtained similar results with *Chenopodium ambrosiodes* on *H. bakeri* eggs and Rosa et al. [28] when evaluating *Persea willdenovii* extracts on gastrointestinal nematodes. The egg hatching rates of *H. polygyrus* had IC_50_ of 0.49 mg/mL and 0.22 mg/mL for the ethanol and aqueous extracts, respectively. These IC_50_ indicate that the aqueous extract is more active for the inhibition of hatching at a 95% confidence interval. According to Adamu et al. [29] crude extracts with IC50 ≥ 6 mg/mL are considered to have weak anthelmintic activity. Therefore, it can be concluded from these criteria of classification that the aqueous (0.49 mg/mL) and ethanol (0.22 mg/mL) extracts showed high ovicidal activity against *H. polygyrus.*

Similar *in vitro* activities were obtained by Rosa et al. [28] where all extracts of *P. willdenovii* inhibited the egg hatching of gastrointestinal nematodes (*p* < 0.05), in a concentration-dependent manner.

Previous studies conducted by Henry et al. [16] have shown that *P. americana* seed extracts contain lipid substances, alkaloids, phenols, and hydro-soluble compounds. From the analysis of the different IC_50_, it is clear that the aqueous extract is more active for the inhibition of hatching at 95% confidence interval. This high ovicidal activity of the aqueous extract may be due to saponins present in their phytochemical composition which is absent in the ethanol extract.

Phytochemical screening revealed the presence of compounds, such as saponine, which is well known for its hatching inhibitory activity [30]. The embryonic development of structures used for hatching, such as the protractible stylet, which is used for hatching may have been inhibited by the plant extract. Furthermore, the extracts may have influenced the osmotic pressure and the shape of the egg by inhibiting the exchange of molecules across the eggshell. However, since the layers of the eggshells are hydrophilic, the aqueous extract can penetrate the eggshell better than ethanol extract. According to Caroline et al. [31], phenolic compounds do not influence egg hatching, which may be due to the eggshell, which is impermeable to phenolic compounds. The observation that the aqueous extract was more active was equally observed by Komtangi et al. [20].

The IC_50_ for aqueous extract was 1.443 mg/mL (95% confidence interval: 86.49–127.4) and 0.97 mg/mL (95% confidence interval: 88.78–120.9), respectively, for the L1 and L2 larvae, and 1.75 mg/mL (95% confidence interval: 90.71–143.7) and 1.34 mg/mL (95% confidence interval: 91.21–129.5) for the ethanol extract, respectively, for the L1 and L2 larvae, respectively. This result shows that L_2_ larva are more sensitive than L1 larva. These findings were similar to earlier reports by Ngouateu et al. [19]. According to these authors, the L1 stage is still having some food or energy reserved since they are just from hatching, and as time goes on, the food reserved and energy gets finished, making them to be more sensitive to the tested compounds. The diffusion and absorption of the extract across the cuticle of *H. polygyrus* may be responsible for the observed larvicidal activity. The negative controls were free of dead larvae proving the fact that the high mortality (100%) rate in the wells containing the extracts was due to the active phytochemicals present in the extract validating its larvicidal activity. High mean inhibitory mobility concentration was observed at 5 mg/mL in a concentration-dependent manner.

Similar observations were made by Payne et al. [32]. According to Payne et al. [32], the active compounds found in the extracts may paralyze the larvae by blocking the post-synaptic receptors. Furthermore, active compounds may diffuse across the cuticle of the larvae, hence increasing the cell permeability which leads to the death of the larvae [33]. The results obtained at this stage were similar to that obtained by Wabo et al. [27] with *C. ambrosiodes* on the different larvae stages of *H. bakeri* and contrary to that obtained by Rosa et al. [28] where a low *in vitro* activity was observed for all the extracts and fractions of *P. willdenovii* leaves on the L_3_ larva of gastrointestinal nematodes.

## 5. Conclusion

This study validates the use of *P. americana* seeds by traditional healers in the fight against helminthiasis. *In silico* and *in vivo* investigations are necessary to confirm their anthelminthic activity. However, validation of a method or technique implies multiple experiments with a deeper statistical analysis.

## Figures and Tables

**Table 1:** Percentage (%) inhibition of hatching, larvae motility, and half maximal inhibitory concentration (IC_50_).

Anthelminthic test	Extract	Concentrations (mg/mL)							Controls		
		0.312	0.625	1.25	2.5	5	IC_50_	95% confidence interval	Levamisole	1.5% DMSO	Ringer 0.9% NaCl
% Inhibition of hatching	Aqueous	49.00 ± 1^e^	62.33 ± 1.45^d^	67.33 ± 1.45^a^	70.33 ± 0.88^a^	78.33 ± 1.67^b^	0.22	74.28–86.18	100.00 ± 0^f^	0.00 ± 0^g^	0.00 ± 0^g^
	Ethanol	45.67 ± 2.33^e^	58.33 ± 1.67^d^	62.33 ± 1.46^c^	67.33 ± 1.45^b^	75.67 ± 1.15^a^	0.49	71.70–92.03	100.00 ± 0^f^	0.00 ± 0^g^	0.00 ± 0^g^
% Inhibition of L1 larva motility	Aqueous	49 ± 2.64^c^	55.33 ± 2.9^bi^	67 ± 1.52^ghi^	75.67 ± 2.84^fh^	85 ± 2.88^a^	1.443	86.49–127.4	100 ± 0^d^	0.00 ± 0^e^	0.00 ± 0^e^
	Ethanol	54.67 ± 3.38^h^	59.67 ± 2.60^g^	71.33 ± 1.86^ci^	78.67 ± 2.02^bi^	89 ± 2.3^a^	1.75	90.71–143.7	100 ± 0^d^	0.00 ± 0^e^	0.00 ± 0^e^
% Inhibition of L2 larva motility	Aqueous	55.67 ± 2.96^dj^	62.33 ± 3.28^cj^	74.67 ± 0.6^bi^	82.67 ± 3.05^ai^	89.33 ± 1.2^abf^	0.97	88.78–120.9	100 ± 0^f^	0.00 ± 0^e^	0.00 ± 0^e^
	Ethanol	62 ± 4.04^dj^	65.00 ± 2.88^chj^	77.00 ± 1.73^bgh^	84.67 ± 0.6^ag^	90.67 ± 1.7^abf^	1.34	91.21–129.5	100 ± 0^f^	0.00 ± 0^e^	0.00 ± 0^e^

a,b,c,d,e,f,g,h,i,j: values carrying the same superscript letter on the same row at different concentrations are not significantly different at *p* ≥ 0.05.

**Table 2:** Qualitative analysis of *P. americana* extracts.

Phytochemicals	Extracts	
	Aqueous	Ethanol
Polyphenol	+	+
Flavonoids	+	+
Anthraquinone	+	+
Triterpenoids	+	+
Saponins	+	−
Alkaloids	+	+
Sterols	−	−

−: absent; +: present.

## Data Availability

All data generated and analyzed are included in this research article.
